# An Inequilateral Triangle: Russia−United States−China in a New Geopolitical Environment

**DOI:** 10.1134/S1019331622130068

**Published:** 2022-12-23

**Authors:** S. M. Rogov

**Affiliations:** Institute for US and Canadian Studies, Russian Academy of Sciences (ISKRAN), Moscow, Russia

**Keywords:** triangle, foreign policy, “chimerica”, nuclear balance, priority challenge, military policy, strategy of “double deterrence”, national defense strategy, military-strategic balance, latticework, concept of “strategic autonomy”

## Abstract

The author offers a general comparative overview of the relations of three key countries—Russia, China, and the United States. A new geopolitical picture of the world is presented with three centers of power. The triangle evolves, changing the potential of each of its sides. The three-party relationships are not equipollent. China is building up its economic, nuclear, and political potential, turning into a peer competitor of the United States on the world stage. Russia competes with the United States only in the military-strategic sphere. That is why US foreign policy today is aimed at “double containment” of both Russia and China. At the same time, Washington seeks to rely on its allies both in Europe, strengthening NATO, and in the Indo−Pacific region, pursuing a “latticework” strategy. This promises a long-term confrontation between the US angle and the Chinese and Russian angles of the triangle.

## INTRODUCTION

I would like to draw attention to several points regarding relations among Russia, China, and the United States. After the Second World War, this triangle has played a very important role in world politics and economics, as well as in the military sphere. Moreover, at present this role is not weakening, but increasing.

The US−China−Russia triangle largely determines the course of events in the world in the 21st century. It includes two economic superpowers; the three largest nuclear states; the 1st, 3rd, and 4th countries in terms of territory; and the 1st, 3rd, and 10th states in terms of population [1].

The sides of the triangle are not equal. China is significantly ahead of Russia in nonmilitary parameters, Washington leads in military spending, and Moscow ranks first in nuclear weapons.

## HISTORY OF THE TRIANGLE

During the First Cold War, relations in the triangle developed in a zigzag fashion. After the victory of the Chinese Communist Party (CCP) with the help of the Soviet Union in the civil war, the United States refused to recognize the People’s Republic of China (PRC). The alliance between Moscow and Beijing against Washington manifested itself in the Korean War, where the American army was opposed by millions of Chinese “people’s volunteers.” The Soviet Union played an important role as a “big brother” in the creation of the political and economic system in the PRC.

However, by the early 1960s, relations between the Communist Party of the Soviet Union (CPSU) and the CCP had deteriorated sharply due to Mao’s unwillingness to support the “fight against the cult of personality” and due to N.S. Khrushchev’s refusal to provide the Chinese with nuclear weapons. The PRC independently created its own nuclear weapons. During the so-called cultural revolution, armed clashes took place on the Soviet−Chinese border.

In 1972, President R. Nixon, realizing that Mao had staked on “the fight against Soviet hegemonism,” visited Beijing, and in 1979 the administration of President J. Carter agreed to official diplomatic recognition of the PRC, depriving the Kuomintang government in Taiwan of the status of the legitimate representative of China. Thus, the American and Chinese corners of the triangle created a structure that resisted the Soviet Union until the late 1980s.

The collapse of the Soviet Union led to the disappearance of the bipolar system of international relations. Washington staked on the consolidation of the unipolar world with the United States as the only superpower. The Soviet Union was no longer among those who could try to catch up, but there were two states that were defeated in World War II, which, under the protection of the American umbrella during the Cold War, turned into powerful economic powers that began to compete with the United States. However, Washington had powerful levers that controlled the military–political independence of Bonn and Tokyo—NATO^1^ and a mutual security treaty.

As a result, the PRC gained broad access to American investments, technologies, and the higher education system. Labor productivity increased significantly. This became one of the main reasons for the extremely rapid pace of development of the Chinese economy over several decades. China came out first in high-tech exports. In the 21st century, it has become a “factory of the world,” employing 236 million people in China’s industry, while the United States employs 34 million, and Russia, 21 million.

China has become the main supplier of household goods to the giant American consumer market. Thus, the economic interdependence of the United States and China arose. At the same time, a gigantic trade deficit has been formed, as well as the American debt to China—more than $1 trillion, which remains to this day.

In 2021, Russia accounted for 0.8% of foreign trade and 0.2% of US foreign investment, ranking 23rd among US trading partners [2] and importing mainly raw materials. Russian−Chinese trade and economic relations are more developed. In 2021, Russia accounted for 27% of Chinese coal imports, 16% of oil, and 8% of gas. At the same time, China accounted for 72% of Russian imports of computers and telecommunications equipment and 56% of semiconductors. China was Russia’s second foreign trade partner after the EU, 18%, while the share of the United States was only 4% [3].

At the beginning of this century, China took second place in the world in terms of GDP and began to approach the United States in many parameters of economic development. In the middle of the past decade, it overtook the United States in terms of GDP at purchasing power parity (PPP).

As a result, the United States lacked a “critical mass” to stop China from becoming a “peer-to-peer” competitor.

This allowed the PRC to use its economic power to begin a large-scale modernization of its armed forces, in no small part due to the purchase and copying of modern Russian weapons. At the same time, the Chinese have deployed many medium-range ballistic missiles in the eastern provinces. The United States withdrew from the INF Treaty, motivating this step by the need to respond to the deployment of missiles of this class by the Chinese, forcing the American fleet to retreat behind the so-called first line of islands, and in the future, the second line of islands.

China seeks to establish a security zone in the coastal seas of the Pacific Ocean and has begun to create an ocean fleet to ensure maritime transit of Chinese export goods and imports of raw materials not only in the Pacific but also in the Indian Ocean. The number of Chinese surface ships and submarines in 2000–2020 increased from 110 to 360 units. This is formally more than that of the US Navy, but in terms of tonnage of warships, the Chinese are three times inferior to the Americans.

The administration of George W. Bush lost control of this situation, mired after September 11, 2001, in an endless war with “Islamofascism.” During this period, the concept of Chimerica appeared—an alliance of two powers under the leadership of the United States. A supporter of this concept was, for example, Zbigniew Brzezinski. However, Beijing rejected such a scenario and began to demonstrate the desire to protect its interests.

Recognizing the challenge from the Chinese giant, the Obama administration announced the transfer of the pivot of American military policy to the Indo-Pacific region. At the same time, Washington tried to create a kind of “common market” for all the states of this region except for China. However, D. Trump unilaterally abandoned this idea and launched an open trade war against China. This has led to some decline in US investment in China and US imports of Chinese goods. Washington began to condemn sharply the Chinese policy in Hong Kong, Tibet, and Xinjiang. Nevertheless, the PRC has overtaken the United States in terms of GDP at PPP, although it continues to lag behind in terms of GDP at the exchange rate.

After the outbreak of the pandemic, American propaganda claimed that the coronavirus was created by the CCP. Biden has generally continued the strategy of containment of the PRC.

As for Russian−Chinese relations, they normalized in the 1990s. Territorial disputes were settled (mainly due to concessions from the Russian Federation) and military tensions were defused in the border areas. Gradually, trade began to improve. Russia has become the main supplier of weapons to China, for many billions of dollars. Joint military exercises have begun to be held.

Perhaps the most important factor was the development of cooperation between Moscow and Beijing in their approach to international affairs, as opposed to Washington. This was clearly manifested in the introduction of a joint draft treaty banning the deployment of weapons in outer space, as well as the creation of multilateral forums such as the RIC, BRICS, and SCO. Although the alliance was not formally concluded, it was proclaimed that “cooperation between Russia and China has no boundaries, our struggle for peace has no boundaries, our desire to maintain security has no limits, our opposition to hegemonism has no limits” [4]. Thus, the term *hegemonism* has been revived, but it is applied not to Moscow but to Washington.

Consequently, the configuration of the triangle has changed again. China and Russia began to balance the United States again, hindering the desire of the Americans to consolidate the unipolar world order. At the same time, Washington will continue to implement the strategy of “double containment” between Moscow and Beijing for the foreseeable future. However, the United States does not seem to have the strength to tackle such a difficult task alone.

## PUBLIC OPINION

Opinion polls in all three countries support the thesis that we are living in a new Cold War (Cold War 2.0).

In recent years, a stable bipartisan anti-Russian and anti-Chinese consensus has developed within the US political elite. The rampant propaganda is unprecedented. Henry Kissinger assessed this as the “demonization” of Russia [5].

Propaganda is also reflected in the sentiments of the American public. The negative assessments of the Russian Federation and the PRC have returned to the 1950s and 1960s and even surpass the stereotypes of that period.

The positive image of Russia that was observed after the end of the first Cold War has disappeared almost without a trace. During the period of perestroika in the Soviet Union in 1989, for the first time, a positive assessment of our country was noted in American public opinion. The last time most respondents gave a positive assessment of our country was in 2011. Since then, negative ratings have steadily increased and, according to Gallup, in 2022 reached the highest level in the history of public opinion polls, 88% on the eve of the start of a special military operation in Ukraine [6]. A Pew Research Center survey showed that the proportion of Americans who consider Russia an “enemy” rose from 41 to 70% from January to March 2022 [7].

Traditionally, the Republican Party has taken a tougher stance against the Soviet Union. This trend continued after the collapse of the Soviet Union. However, after the 2016 presidential election, the Democrats overtook the Republicans in their negative attitude towards the Russian Federation. After Trump came to power, the Republicans continued to somewhat lag behind the Democrats in this indicator. However, now the parties have equalized in support of anti-Russian views. It seems that this situation will continue for a long time.

A similar picture is observed in Russia. Here, too, during the years of perestroika, the negative attitude towards the United States began to change to a positive one. This continued, according to the Levada Center,^2^ until the NATO intervention in Yugoslavia. Later, the war in South Ossetia and the first Maidan in Ukraine played a negative role. However, the situation finally changed in 2014. The new Cold War consolidated the negative image of the United States. In the spring of 2022, 75% of respondents believed that the United States is unfriendly to Russia.

As for China, the secret diplomacy of Kissinger paved the way for Nixon’s visit to Beijing in 1972 and then for the normalization of relations between the United States and China. After the establishment of diplomatic relations in 1979, 66% of Americans viewed China favorably. The development of US−Chinese cooperation to contain the Soviet Union brought this figure to 72%. However, the events on Tiananmen Square led to a more than twofold reduction in positive attitudes towards China, 34%. Subsequently, positive estimates remained at the level of 40–45% for many years, until they dropped to 21% in 2021. Now the negative attitude towards China has reached 79%, which is even somewhat worse than towards Russia [6].

Ninety-two percent of Americans believe that the partnership between Russia and China is a “serious problem” for America, and 64%, that “China’s power and influence pose a serious threat” [8]. However, only 25% of those surveyed consider China an “enemy” (12% Democrats and 45% Republicans), and 62%, only a “rival.”

Thus, the rampant propaganda has led to the fact that stable negative stereotypes have developed in the public mind of the United States regarding Russia and China. The same thing also happened with respect to the United States in the mood of the Russian and Chinese public. Historical experience shows that it will take many years and even decades to revise these stereotypes, which will also be reflected in the approach of political circles.

## DOCTRINAL INNOVATIONS OF THE BIDEN ADMINISTRATION

Already in the very first doctrinal document of the Biden administration, published in March 2021, the Chinese and Russian angles of the triangle were assessed: “Both Beijing and Moscow have invested heavily in efforts meant to check US strengths and prevent us from defending our interests and allies around the world.” However, important differences were recognized: “China, in particular, has rapidly become more assertive. It is the only competitor potentially capable of combining its economic, diplomatic, military, and technological power to mount a sustained challenge to a stable and open international system.” In this regard, it is proclaimed [9, p. 20]:

We will ensure that America, not China, sets the international agenda, working alongside others to shape new global norms and agreements that advance our interests and reflect our values. By bolstering and defending our unparalleled network of allies and partners, and making smart defense investments, we will also deter Chinese aggression.

Russia is not recognized as such a rival, although it is stated that it “remains determined to enhance its global influence and play a disruptive role on the world stage” [9, p. 8].

In April 2022, the US Department of Defense published a doctrinal document, the National Defense Strategy. This is the short version, just two pages, and it is impossible not to read it carefully without concluding that the United States sees China as its number one adversary. Moreover, China is mentioned three times on these two pages and described as a priority challenge for the United States. Russia is mentioned twice and described as an acute threat [10]. Apparently, this is due to the events in Ukraine and the military operation that Russia is conducting there. However, it is clear that China is perceived as an adversary in the economic, political, ideological, and military spheres, whereas Russia is considered primarily a direct military threat to the United States, and the reason for this is the presence of an impressive nuclear arsenal in Moscow. However, in economic terms, Russia is not seen as a competitor to the United States.

The Biden administration has made no secret of its intention to defeat Russia in a special military operation in Ukraine. Washington has almost completely frozen diplomatic contacts with Moscow and hinders Russian−Ukrainian peace talks. The United States and its allies have provided Kyiv with financial and military assistance that exceeds the Ukrainian state budget. At the same time, the West unleashed a real economic war against our country, having worked out in advance some measures that had never been used before in peacetime.

The unprecedented economic sanctions imposed by the United States and its allies against Russia will lead, according to some experts, to a reduction in Russian GDP by 10% in 2022. The sanctions were described as an attempt to destroy the Russian economy, but this did not happen, although it led to serious socioeconomic consequences for Russia. It will take about ten years to bring our economy back to the 2021 level.

On March 26 of this year in Warsaw, Biden said that the Russian president, whom he called a “war criminal” after the start of the special military operation, “should not remain in power” and actually supported “regime change in Russia” [11]. Although the State Department later denied that this was a US goal. Clearly what is happening is similar to Washington’s attempt to change the regime in Moscow.

The second aspect relates to the notion promoted by Graham Alison about the history of great power rivalry, and his conclusion was that a clash between China and the United States is almost inevitable (“Thucydides trap”) [12]. According to the concept of the ancient Greek historian Thucydides, during the rivalry between the great powers of Athens and Sparta, a military conflict was almost inevitable. This perception is shared by many American experts who are trying to figure out how to prevent such an outcome.

The next aspect relates to the idea that, if Russia can be successful and achieve its goals in Ukraine, this will encourage China to use military force to reunite with Taiwan. There is a fierce debate in the United States about how to protect Taiwan from the PRC by creating a strategy that will deny a Chinese victory. This approach is most aggressively promoted by Elbridge Colby [13]. Such a denial strategy includes several elements, providing Taiwan with sufficient military equipment, as well as building up US strategic and nonstrategic nuclear forces and missile defense. All this should deprive China of the ability to launch a preemptive strike against US aircraft carrier groups and military bases in the Western Pacific.

Finally, there is a dispute in the American expert community about how to destroy a potential Russian−Chinese alliance that would become a counterbalance to the United States. Some American experts suggest that the United States should carry out the Kissinger maneuver in reverse, that is, establish partnerships with Russia to contain China [14].

In reality, however, the Biden administration is pursuing a completely different policy: its actions against Russia are pushing the latter towards closer relations with China. Recently, there have been speculations that there is an opportunity to push China away from supporting Russia with the help of threats of imposing secondary economic sanctions against Chinese companies. Thus, if China continues to develop economic relations with Russia, then it will be punished by economic sanctions from the United States and its allies. However, the idea of the PRC breaking away from Russia is disputed by some experts because they point to the economic interdependence both between China and the United States and between China and Europe. They state that sanctions against China would be counterproductive.

Some American experts argue that the current concern about the Russian threat should not dampen attention to China as the main long-term priority of American policy in the 21st century. As a recent report from the Congressional Research Service highlights, “The key issues observers are currently debating include how much priority US defense planning should give to Europe (to deter or respond to Russian actions) versus the Indo−Pacific (to deter China),” specifically defining “how the US response to Russia’s invasion of Ukraine might influence Chinese calculations regarding potential actions it might take toward Taiwan.” These discussions may “lead to changes in the US grand strategy or defense strategy, and/or the size of the US defense budget” [15].

## NUCLEAR BALANCE

Comparison of the military–strategic balance shows that the Russian Federation remains a nuclear superpower and still maintains approximate parity with the United States. There are no official data, but, according to SIPRI, each side has 6000 nuclear warheads, which is 15–20 times more than China has [16]. Russia and the United States continue to comply with the START-3 Treaty, signed in 2012 and extended until 2026 [17]. As of March 1, this year, the United States had 1515 nuclear warheads deployed on 686 delivery vehicles (ICBMs, SLBMs, heavy bombers), and Russia had 1474 and 761, respectively [18].

At the same time, it is believed that Moscow significantly outnumbers Washington in nonstrategic nuclear warheads [19]. Experts from the Federation of American Scientists claim that the United States has only 200 such warheads [20], while Russia has about 2000 [21].

A new generation of US cruise, ballistic, and hypersonic missiles will likely begin to be deployed in 2023 not only against China but also near Russian borders in Europe, for example, in the Baltic and Poland. This will allow American missiles with a short flight time to hit many strategic targets on the territory of the Russian Federation.

In 2022, the United States broke off negotiations with Russia on strategic stability and the development of a new treaty to replace START-3. Considering that earlier Washington unilaterally withdrew from the ABM, INF, and open skies treaties, there is the prospect of a complete and irreversible collapse of the arms control regime that has been in place for several decades.

It should be noted that the American allies, who for many years declared their support for arms control, followed the lead of the United States and supported the rupture of the above agreements. There are no intelligible proposals from the Europeans.

At the same time, there is great concern in the United States, which relates to the buildup of Chinese nuclear forces and the construction of several hundred silo launchers (silos) for ICBMs, which, according to American experts, China is conducting. This will allow the PRC to acquire even more strategic nuclear weapons in the next few years, and in ten or 20 years to catch up and even surpass the United States and Russia in this indicator. Thus, the prospect of a trilateral strategic arms race in the second quarter of this century is emerging.

A possible rapid buildup of China’s nuclear potential is hardly in the interests of the Russian Federation. It is no coincidence that the extreme right circles of the United States are already calling for a withdrawal from START to immediately resume the buildup of the nuclear arsenal, to abandon parity with Russia, and prevent parity with China.

## NATO’S NEW STRATEGY: COALITION STRATEGY

The coalition strategy of the Biden administration was primarily aimed at overcoming the crisis in the North Atlantic Alliance, which was provoked by Trump’s rhetoric. To a certain extent, this was done, and Washington was able to restore its leadership in NATO. This gives the United States the opportunity to mobilize the resources of its European allies to contain Russia.

In the economic sphere, Europe is practically not inferior to the Americans: Europeans account for 15% of world GDP and 12% of industrial production, 24% of exports and 25% of R&D spending. Formally, these figures enable the European Union to claim the role of another superpower. However, the European Union does not have sovereignty for independent action contrary to the position of the “senior partner,” the United States. Therefore, the Biden administration was able to coordinate the economic sanctions of the West against the Russian Federation and the supply of weapons and the provision of financial assistance to Ukraine.

In addition, the European members of NATO have 57% of military personnel and 68% of tanks, 61% of armored combat vehicles, 69% of large-caliber artillery systems, 57% of large surface ships, and 54% of submarines that the North Atlantic Alliance possesses [22]. This significantly exceeds the number of Russian troops and conventional weapons in Europe.

Attempts to implement the concept of “strategic autonomy” for Europe were blocked by Washington, which achieved confirmation of the dominant role of NATO under the US leadership. Under the American auspices, a new doctrinal document was prepared—NATO 2022 Strategic Concept [23].

The document announced the rejection of the previously proclaimed partnership with our country and stated that “the Russian Federation is the most significant and direct threat to Allies’ security and to peace and stability in the Euro−Atlantic area” [23, p. 4]. In this regard, the need is proclaimed to “strengthen deterrence and defense for all Allies.” The United States announced its intention to increase its troops in Europe to 100 000 people, and the total grouping of NATO troops near the Russian borders will grow to 300 000 [24].

The new strategy proclaims that NATO allies are committed to deploying additional strong combat-ready forces on the ground on our eastern flank, building up from existing battlegroups to brigade-level units where and when the need arises, based on convincing, rapidly available reinforcements, prepositioned equipment, and an improved command and control system. We welcome cooperation between frame and host countries in strengthening command and control forces, including the establishment of division-level structures. NATO welcomes initial proposals for a new NATO force model that will strengthen and modernize NATO’s force structure and resources for our next-generation military plans. The alliance will improve its collective defense exercises to be ready for high-intensity operations in various areas and to ensure the strengthening of any NATO member country in a short time. All these steps will significantly enhance NATO’s deterrence and forward defense capabilities [23].

The Madrid summit supported the admission of Sweden and Finland to the North Atlantic Alliance. The line of confrontation between NATO and Russia will more than double. This may lead to increased tension in the Baltic and Northern regions. The current wave of NATO enlargement opens the door for the next invitation to other members of the alliance.

**Fig. 1.**
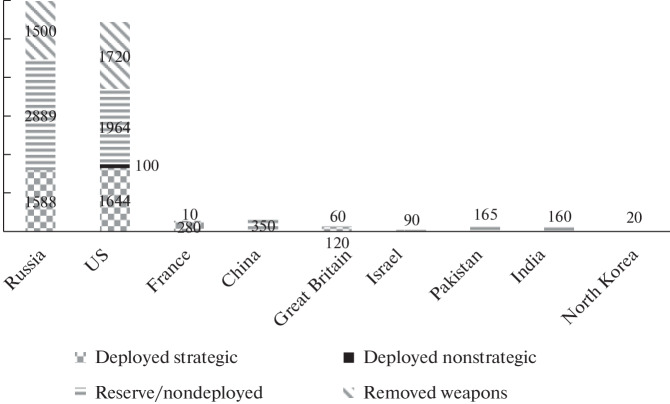
Estimated global nuclear arsenal in 2022. Based on https://fas.org/issues/nuclear-weapons/status-world-nuclear-forces/.

**Table 1.** Share of triangle countries in global indicators in 2021–2022, %

	Population	GDP (PPP)	Exports	R&D	Defense spending	Nuclear weapons
United States	4.3	15.7	10.1	24.5	39	42.7
China	18.4	18.6	12.8	25.5	14	2.7
Russia	1.9	3.1	1.7	2.5	3.4	47

https://www.imf.org/en/Publications/WEO/Issues/2022/04/19/world-economic-outlook-april-2022; https://www.rdworldonline.com/2021-global-rd-funding-forecast-released/; https://www.iiss.org/publications/the-military-balance; https://fas.org/issues/nuclear-weapons/status-world-nuclear-forces/.

**Table 2.** Share of EU and NATO countries in global indicators, %

	Population	GDP (PPP)	Exports	R&D	Defense spending	Nuclear weapons
United States	4.3	15.7	10.1	24.5	39	42.7
China	18.4	18.6	12.8	25.5	14	2.7
Great Britain	0.9	2.3	3.1	2.1	2.5	1.8
European Union	4.4	12.0	26.0	17.4	15	2.5
NATO	8.8	27.9	36.4	42.5	53	46.8

https://www.imf.org/en/Publications/WEO/Issues/2022/04/19/world-economic-outlook-april-2022, https://www.rdworldonline.com/2021-global-rd-funding-forecast-released/, https://www.iiss.org/publications/the-military-balance, https://fas.org/issues/nuclear-weapons/status-world-nuclear-forces/.

**Table 3.** Share of AUKUS, Quad, PBP countries, and China in global indicators, %

	Population	GDP (PPP)	Exports	R&D	Defense spending	Nuclear weapons
United States	4.3	15.7	10.1	24.5	39	42.7
China	18.4	18.6	12.8	25.5	14	2.7
Japan	1.6	3.8	3.3	7.5	2.5	0
India	18.1	7.0	2.4	3.8	3.7	1.3
Great Britain	0.9	2.3	3.1	2.1	2.5	1.8
Australia	1.2	1.9	2.1	1.2	1.5	–
AUKUS	6.4	19.9	15.3	26.7	42.9	45.8
Quad	24.7	44.8	18.9	39.1	49.1	43.9
PBP	8.0	23.7	18.5	34.2	45.4	45.8

https://www.imf.org/en/Publications/WEO/Issues/2022/04/19/world-economic-outlook-april-2022, https://www.rdworldonline.com/2021-global-rd-funding-forecast-released/, https://www.iiss.org/publications/the-military-balance, https://fas.org/issues/nuclear-weapons/status-world-nuclear-forces/.

**Table 4.** The share of the BRICS countries in global indicators, %

	Population	GDP (PPP)	Exports	R&D	Defense spending	Nuclear weapons
China	18.4	18.6	12.8	25.5	14	2.7
India	18.1	7.0	2.4	3.8	3.7	1.3
Russia	1.9	3.1	1.7	2.5	3.4	47
RSA	0.8	0.6	0.5	0.3	0.1	0
Brazil	2.8	2.4	1.1	1.6	0.9	0
BRICS	42	31.7	18.5	33.7	22.1	51

https://www.imf.org/en/Publications/WEO/Issues/2022/04/19/world-economic-outlook-april-2022, https://www.rdworldonline.com/2021-global-rd-funding-forecast-released/, https://www.iiss.org/publications/the-military-balance, https://fas.org/issues/nuclear-weapons/status-world-nuclear-forces/.

**Table 5.** The triangle and international structures as a percentage of global performance

	Population	GDP (PPP)	Exports	R&D	Defense spending	Nuclear weapons
United States	4.3	15.7	10.1	24.5	39	42.7
China	18.4	18.6	12.8	25.5	14	2.7
Russia	1.9	3.1	1.7	2.5	3.4	47
European Union	4.4	12.0	26.0	17.4	15	2.5
NATO	8.8	27.9	36.4	42.5	53	46.8
AUKUS	6.4	19.9	15.3	26.7	42.9	45.8
Quad	24.7	44.8	18.9	39.1	49.1	43.9
BRICS	42	31.7	18.5	33.7	22.1	51
PBP	8.0	23.7	18.5	34.2	45.4	45.8

https://www.imf.org/en/Publications/WEO/Issues/2022/04/19/world-economic-outlook-april-2022, https://www.rdworldonline.com/2021-global-rd-funding-forecast-released/, https://www.iiss.org/publications/the-military-balance, https://fas.org/issues/nuclear-weapons/status-world-nuclear-forces/.

The new NATO document mentions the PRC for the first time, although the Asia−Pacific region (APR) is not included in the geographic scope of the North Atlantic Alliance. The document notes “systemic challenges” posed by China, confronting “our interests, security, and values” and “striving to subvert the rules-based international order” [23, p. 5].

Two points of the strategic concept are devoted to China. At the same time, such harsh language is not applied to Beijing the way it is to Moscow.

For the first time, some US Pacific allies were invited to the NATO summit. In this regard, the document notes that the participation of partners from the APR, along with other partners, has demonstrated the value of our cooperation in countering common security challenges [23, p. 11].

The strategy announces, “The Indo−Pacific is important for NATO, given that developments in that region can directly affect Euro−Atlantic security. We will strengthen dialogue and cooperation with new and existing partners in the Indo–Pacific to tackle cross-regional challenges and shared security interests” [23, p. 11].

This corresponds to the concept of a triangle and indicates Washington’s desire to involve NATO in the confrontation between the United States and China. However, European allies show little enthusiasm for a confrontation with the PRC, which is their biggest trading partner. Perhaps the only exception was Great Britain, which, under B. Johnson, again tried to claim a global role.

## LATTICEWORK COALITIONS

Washington does not have a powerful military bloc in the Pacific like NATO. However, it should not be forgotten that the United States has bilateral mutual security treaties with Japan and South Korea, which host military bases where approximately 40 000 US troops are stationed. However, this is clearly not enough to contain China. In addition, relations between Tokyo and Seoul are very difficult.

Under these conditions, the Biden administration decided to create several coalition formations in the Indo-Pacific region with a predominance of the “Anglo-Saxon component.” Former Undersecretary of State Christopher Ford called this innovation “latticework” [25].

The first example of such “latticework” was the AUKUS grouping, which in August 2021 included the United States, Great Britain, and Australia under the pretext of cooperation in the creation of nuclear submarines [26].

The largest coalition is the Quad, consisting of the United States, Australia, Japan, and India. The first summit of the Quad was held in autumn 2021. This is the only group that formally surpasses China in terms of population by 25%, GDP by 45%, and exports by 19% [27].

Finally, in the summer of 2022, the creation of the Partners in the Blue Pacific (PBP) was announced, which included five countries, including AUKUS members, as well as New Zealand and Japan. This is due to the opposition to the attempts of the PRC to settle in the Solomon Islands in the South Pacific Ocean [28].

Thus, the United States is trying to encircle China. However, they have failed to involve both India and Japan in one coalition at the same time. In addition, none of the groups included Canada and South Korea. Apparently, the Biden administration will continue its efforts to create the broadest possible unified anti-Chinese coalition.

In turn, Moscow and Beijing are also seeking to acquire partners. The BRICS group mentioned above formally has a very impressive potential: 42% of the population, 32% of GDP, and 19% of world exports [1]. In fact, the BRICS group is not in a position to act as a single alliance like NATO.

In addition, the contradictions between China and India are too strong, including the long-standing territorial conflict. Moreover, India has joined the Quad, which is clearly anti-Chinese.

The Russian Federation has common interests with China, but they do not always fully coincide. This, in particular, concerns problems such as Crimea and Taiwan.

It is important to note the emergence of new multinational formats. They do not have the same potential.

## CONCLUSIONS

Some of these structures will not last long, others may have a long way to go and may become real economic and military alliances.

The Congressional Research Service presented its interpretation of the triangle. It states that “the renewed great power rivalry is not a bipolar situation (like the Cold War) or a unipolar situation (like the post-Cold War era), but a situation characterized in substantial part by renewed competition among three major world powers—the United States, China, and Russia.” This situation is described as Cold War 2.0 [15].

I think that we are witnessing a new geopolitical situation where changes can happen quite quickly. It seems to me that the outcome of the special military operation in Ukraine will largely determine the future of relations in the Russia−United States−China triangle.

## Footnotes

1 According to the classic formula of NATO’s first Secretary General Lord Ismay, NATO’s goal is “to keep America in [Europe], Germany down, and the Soviet Union out [of Europe].”

2 Recognized as a foreign agent.

